# Elucidating the dynamics of polypeptide hormones in the physiological and preeclampsic placental trophoblast cells across gestation at single-cell level

**DOI:** 10.1093/lifemedi/lnad003

**Published:** 2023-02-01

**Authors:** Hao Wu, Chenxiang Luo, Yin Rong, Ruoxuan Yu, Yan Zhao, Long Yan, Hongmei Wang

**Affiliations:** State Key Laboratory of Stem Cell and Reproductive Biology, Institute of Zoology, Chinese Academy of Sciences, Beijing 100101, China; Institute for Stem Cell and Regeneration, Chinese Academy of Sciences, Beijing 100101, China; Beijing Institute for Stem Cell and Regenerative Medicine, Beijing 100101, China; Department of Obstetrics and Gynecology, Reproductive Medical Center, The First Affiliated Hospital of Sun Yat-sen University, Guangzhou 510080, China; State Key Laboratory of Stem Cell and Reproductive Biology, Institute of Zoology, Chinese Academy of Sciences, Beijing 100101, China; Institute for Stem Cell and Regeneration, Chinese Academy of Sciences, Beijing 100101, China; Beijing Institute for Stem Cell and Regenerative Medicine, Beijing 100101, China; University of Chinese Academy of Sciences, Beijing 100049, China; State Key Laboratory of Stem Cell and Reproductive Biology, Institute of Zoology, Chinese Academy of Sciences, Beijing 100101, China; Institute for Stem Cell and Regeneration, Chinese Academy of Sciences, Beijing 100101, China; Beijing Institute for Stem Cell and Regenerative Medicine, Beijing 100101, China; University of Chinese Academy of Sciences, Beijing 100049, China; State Key Laboratory of Stem Cell and Reproductive Biology, Institute of Zoology, Chinese Academy of Sciences, Beijing 100101, China; Institute for Stem Cell and Regeneration, Chinese Academy of Sciences, Beijing 100101, China; Beijing Institute for Stem Cell and Regenerative Medicine, Beijing 100101, China; University of Chinese Academy of Sciences, Beijing 100049, China; State Key Laboratory of Stem Cell and Reproductive Biology, Institute of Zoology, Chinese Academy of Sciences, Beijing 100101, China; Institute for Stem Cell and Regeneration, Chinese Academy of Sciences, Beijing 100101, China; Beijing Institute for Stem Cell and Regenerative Medicine, Beijing 100101, China; State Key Laboratory of Stem Cell and Reproductive Biology, Institute of Zoology, Chinese Academy of Sciences, Beijing 100101, China; Institute for Stem Cell and Regeneration, Chinese Academy of Sciences, Beijing 100101, China; Beijing Institute for Stem Cell and Regenerative Medicine, Beijing 100101, China; University of Chinese Academy of Sciences, Beijing 100049, China

## Dear Editor,

Polypeptide hormones are essential for establishment and maintenance of pregnancy, maternal adaptation to pregnancy and fetal development. Altered polypeptide hormones in maternal serum were shown in multiple feared pregnancy complications, such as preeclampsia (PE), which is characterized by pregnancy-specific hypertensive and multisystem disorders. The multinucleated syncytiotrophoblast (STB) is considered to be the main source for most of the known polypeptide hormones, such as human chorionic gonadotropin (hCG), human placental lactogen (hPL), human somatomammotropic hormone (hCS), and placental growth hormone (GH). However, the other two major placental cell types, proliferative mononucleated cytotrophoblast cell (CTB) and invasive extravillous trophoblast cell (EVT), were also found to be able to produce various polypeptide hormones based on their transcriptomic characteristics obtained from a single-cell RNA sequencing (scRNA-seq) of the human placenta in first trimester [1]. This study also showed a surprising 102 polypeptide hormone-encoding genes were expressed by early placental cells, suggesting that different polypeptide hormones may play various roles during placental development. Accordingly, a comprehension of the polypeptide hormones secreted by different placental cells in the normal and pathological placentas across gestation is very important but lacking.

**Figure 1.**
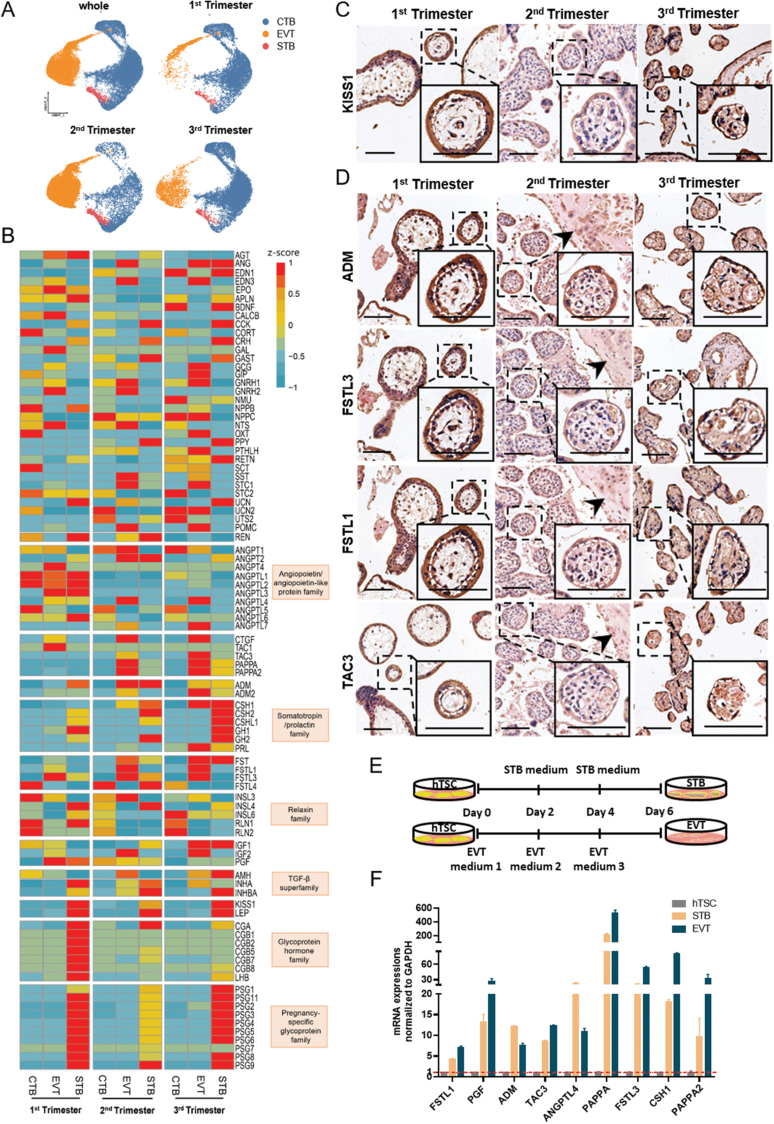
**The expression of polypeptide hormone-encoding genes in three types of trophoblast cells throught pregnancy. **(A) UMAP diagram showing three types of trophoblast cells in human placenta during the first, second, and third trimester (*n* = 11 placentas from first trimester, 2 placentas from second trimester and 2 placentas from third trimester). CTB, cytotropgoblast cell; STB, syncytiotrophoblast; EVT, extravillous trophoblast cell. (B) Heatmap illustrating relative expression (z-score) of 91 polypeptide hormone-encoding genes in three types of trophoblast cells during the first, second, and third trimester. (C) Immunohistochemistry staining for KISS1 in the placental villi from first, second, and third trimester (*n* = 3). White dotted lines separate the inner CTBs from the outer STBs. Scale bars, 100 μm. (D) Immunohistochemistry staining for ADM, FSTL3, FSTL1, and TAC3 in the placental villi from first, second, and third trimester and in the decidua from second trimester (*n* = 3). White dotted lines separate the inner CTBs from outer STBs. Black arrowhead showing the decidua from second trimester. Scale bars, 100 μm. (E) Schematic depiction of the differentiation of hTSCs. (F) Quantification of highly expressed polypeptide hormone-encoding genes in hTSC-derived STBs and EVTs by RT-PCR. Data are shown as mean ± S.D. (*n* = 3).

To systematically understand the polypeptide hormones secreted by trophoblast cells throughout gestation, we analyzed the expression of polypeptide hormone-encoding genes in the scRNA-seq data of 11 placentas from first trimester (7 published by us [2] and 4 published by Vento-Tormo, et al. [1]), 2 placentas from second trimester and 2 placentas from third trimester. The three types of trophoblast cells were identified according to the expression of reported markers [2]: CTB (CDH1^+^, EGFR^+^, and HLA-G^−^), EVT (HLA-G^+^, and MMP2^+^), and STB (CGA^+^, CGB^+^, and CSH1^+^) (Fig. 1A). A total of 91 polypeptide hormone-encoding genes was found in all types of trophoblast cells throughout gestation by microarray assay (Fig. 1B). We found that besides well-known polypeptide hormone-encoding genes in STBs, such as the CGB family and PSG family genes, KISS1, which encodes kisspeptin and is considered to play an important role in embryo implantation and placentation, was specifically highly expressed by STBs [3]. By immunohistochemical staining (IHC), we further confirmed that STBs were the major source of kisspeptins (Fig. 1C). Some other polypeptide hormone-encoding genes were also found to be highly expressed by STBs, such as the angiopoietin/angiopoietin-like protein family genes, including ANGPTL1, ANGPTL2, and ANGPTL6, and the somatotropin/prolactin family genes, including CSH1, CSH2, CHL1, GH1, GH2, and PRL (Fig. 1B). Notably, EVTs expressed polypeptide hormone-encoding genes, including those involved in angiogenesis, including ADM, CSH1, FSTL1, PGF, TAC3, and ANGPTL4, and trophoblast invasion, including FSTL3, PAPPA, and PAPPA2, which are highly correlated with the functions of EVTs in remodeling the maternal spiral arteries and invasion in the maternal decidua (Fig. 1B). CTBs expressed polypeptide hormone-encoding genes, such as CORT, ANGPTL5, UTS2, and RNL2, and the polypeptide hormones encoded by CORT, UTS2, and RNL2 were reported to have altered serum concentrations in PE patients (Fig. 1B). Moreover, we performed IHC in the placental villi from first, second, and third trimesters, and the decidua from second trimester, and showed that the proteins encoded by ADM, FSTL3, FSTL1, and TAC3 were expressed by all three types of trophoblast cells (Fig. 1D).

To further confirm the changes of polypeptide hormones during trophoblast cell differentiation, we used human trophoblast stem cells (hTSCs) to simulate the differentiation of trophoblast cells [4]. We induced the hTSCs into STBs and EVTs as we reported previously (Fig. 1E) [5], and demonstrated that several polypeptide hormone-encoding genes were up-regulated after differentiation of hTSCs, such as FSTL1, PGF, ADM, TAC3, ANGPTL4, PAPPA, FSTL3, CSH1, and PAPPA2, which was consistent with the results of scRNA-seq, suggesting that hTSC-based trophoblast cells served as promising models to indicate the changes of polypeptide hormones with trophoblast cell differentiation (Fig. 1F).

Altered polypeptide hormones in the circulation of PE patients have been extensively reported. However, whether the abnormal hormones arise directly from pathological placenta remains unclear [6]. A systematic study on the expression pattern of polypeptide hormones in the placentas from PE patients will help to understand the etiology of PE and find better biomarker to detect PE, while the study is lacking.

**Figure 2.**
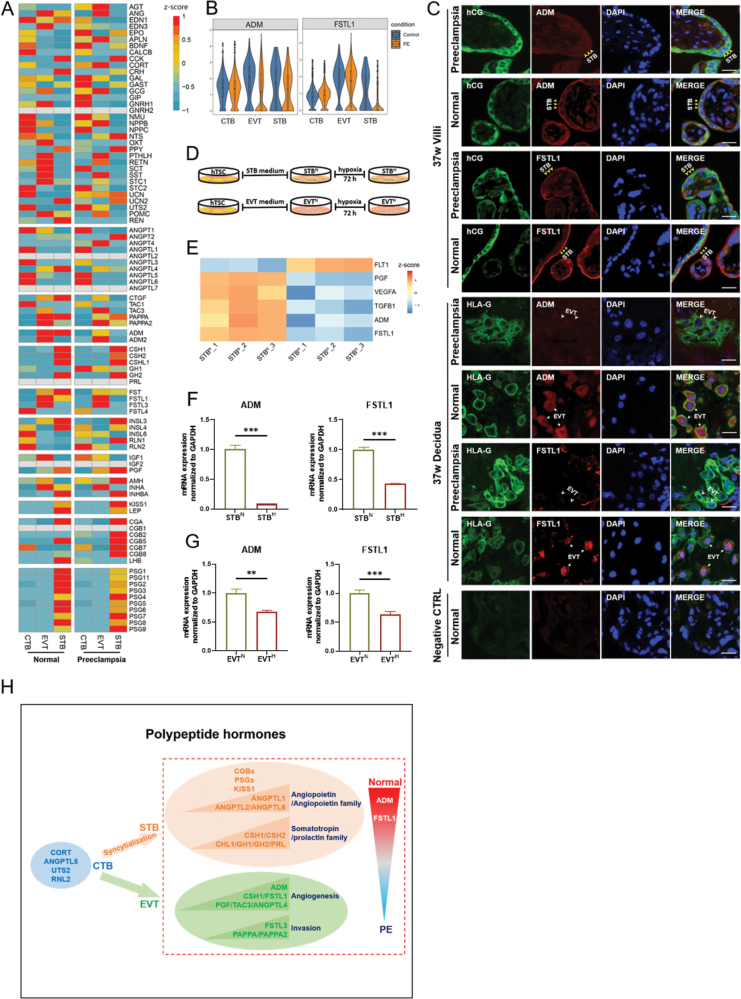
**Simulating the expression of polypeptide hormone-encoding genes under physiological and PE conditions using hTSC-derived trophoblast cells.**(A) Heatmap illustrating relative expression (z-score) of 91 polypeptide hormone-encoding genes in three types of trophoblast cells from normal pregnant women and PE patients. (B) Violin plots showing a decreased expressions of ADM and FSTL1 in both STBs and EVTs of PE placentas (*n* = 2) compared to normal placentas (*n* = 2). (C) Immunofluorescence staining for ADM, FSTL1, hCG, and HLA-G in the placental villi and decidua from the 37 weeks normal pregnant women and 37 weeks PE pregnant women (*n* = 3). Yellow arrows showing the STBs with highly expression of ADM/FSTL1 from normal pregnant women; yellow arrowheads showing the STBs with low expression of ADM/FSTL1 from PE pregnant women. White arrows showing the EVTs with highly expression of ADM/FSTL1 from normal pregnant women; white arrowheads showing the EVTs with no ADM/FSTL1 expression from PE pregnant women. Negative control (CTRL) showing the immunofluorescence staining results without adding primary antibody. Scale bars, 20 μm. (D) Schematic depiction of hypoxia treatments of hTSCs-derived STBs and EVTs. STB^N^, STB cultured in normoxia. STB^H^, STB cultured in hypoxia. EVT^N^, EVT cultured in normoxia. EVT^H^, EVT cultured in hypoxia. h, hours. (E) Heatmap illustrating the expression of indicated differentially expressed genes in normal (STB^N^) and hypoxia (STB^H^) treated STBs. (F) Quantification of ADM and FSTL1 mRNA expression in normoxic and hypoxic STBs by RT-PCR (*n* = 3). Graph showing the expression level relative to the geometric mean of the housekeeping gene GAPDH. Data are shown as mean ± S.D., ***P < 0.001 (relative to levels in STB^N^). (G) Quantification of ADM and FSTL1 mRNA expression in normoxic and hypoxic EVTs by RT-PCR (*n* = 3). Graph showing the expression level relative to the geometric mean of the housekeeping gene GAPDH. Data are shown as mean ± S.D., **P < 0.01, ***P < 0.001 (relative to levels in EVT^N^). (H) Graphical summary of this study.

To characterize the changes of polypeptide hormones in the trophoblast cells of PE placenta, we analyzed the scRNA-seq data of 2 normal placentas and 2 PE placentas (published by Zhou et al. [7]). We found polypeptide hormone-encoding genes, including POMC, STC1, ADM, FSTL1, and ANGPTL4 were decreased in STBs (Fig. 2A). Increased GAL and INSL4 in CTBs, and increased CTGF and TAC3 and decreased ADM and FSTL1 in EVTs were also found in the PE placentas (Fig. 2A). Notably, the expressions of ADM and FSTL1 decreased in both STBs and EVTs of the PE placenta compared to those in normal controls (Fig. 2A and 2B). ADM encodes adrenomedullin, a 52-amino acid peptide hormone known to be highly expressed in vessels and highly vascularized organs. During normal pregnancy, adrenomedullin in maternal circulation has been reported to be increased steadily with gestational age and decreased rapidly after delivery, which was known to be important in vasodilation and angiogenesis [8]. We confirmed a decreased adrenomedullin in the STBs and EVTs of PE placenta by immunofluorescence staining (IF) (Fig. 2C). FSTL1 encodes Follistatin-like 1 known to have an intensive role on pro-angiogenesis, inflammation, cell proliferation, and migration in cardiovascular and other systems [9]. IF showed that follistatin-like 1 was significantly decreased in the STBs and EVTs of the PE placenta (Fig. 2C).

The PE placenta was known to undergo hypoxia stress which resulted from impaired spiral artery remodeling. The PE placentas were characterized by increased sFlt-1 and decreased PIGF, VEGF, and TGF-β. We verified the changes of polypeptide hormone-encoding genes found in PE placentas using a hypoxia-treated hTSC-derived trophoblast cell model. In this model, we treated hTSC-derived STBs with hypoxia for 72 h (Fig. 2D) and performed bulk RNA-seq. Following hypoxia treatment, the pro-angiogenic genes, including PIGF, VEGFA, and TGFB1, were reduced, while the expression of FLT1 was increased, which was consistent with the changes in primary trophoblast cells of PE placenta (Fig. 2E). Using this model, we further confirmed that the expression of ADM and FSTL1 were significantly decreased in both hypoxia-treated STBs (STB_H_) and EVTs (EVT_H_) by RT-PCR (Fig. 2E–G).

In conclusion, we profiled the expression of polypeptide hormone-encoding genes in normal trophoblast cells throughout gestation and in PE trophoblast cells, which deepened our understanding of the polypeptide hormone function during normal and PE pregnancy (Fig. 2H).

## Research limitation

One limitation of our study is the omission of some STB of large size by 10× Genomics, which can be improved by single nuclear sequencing. Another limitation in this study is that we showed the transcriptional changes of the polypeptide hormone-encoding genes in the placental trophoblast cells of PE patients, which could not fully represent the changes of polypeptide hormones of PE patients in secretion level. It is necessary to validate the correlation between altered polypeptide hormones and PE by collecting maternal peripheral blood of PE patients for detection.

## Supplementary Material

lnad003_suppl_Supplementary_Material
