# Supplementary material for: Elucidating the dynamics of polypeptide hormones in the physiological and preeclampsic placental trophoblast cells across gestation at single-cell level
Source: Life Med. 2023 Feb 1;2(3):lnad003. doi: 10.1093/lifemedi/lnad003 (PMC11749468; doi:10.1093/lifemedi/lnad003)
Supplement: lnad003_suppl_Supplementary_Material [file lnad003_suppl_Supplementary_Material.docx]

**Supplementary Information**

**Elucidating the dynamics of polypeptide hormones in the physiological and preeclampsic placental trophoblast cells across gestation at single-cell level**

**Material and methods**

**Ethical considerations**

This research was approved by the Ethics Committee of Beijing Obstetrics and Gynecology Hospital, Capital Medical University (research license number: 2022-KY-074-01). Signed informed consent was obtained from all pregnant women who donated their placentas. Samples were used according to standard experimental protocols approved by the Ethics Committee of the Institute of Zoology, Chinese Academy of Sciences. Placentas were collected for performing single-cell RNA sequencing and for immunohistochemistry and immunofluorescence staining.

**Single-cell RNA-seq data pre-processing and quality control**

The paired-end reads of Smart-seq2 data were processed using the custom scripts of Drop-seq_tools-2.0.0. For read 2, bases 1 to 8 were tagged with cell-barcode “XC,” and bases 9 to 16 were tagged with UMI “XM.” After removing the adaptors and TSO sequences and poly(A) sequences, STAR

aligner was used to align the filtered reads to the human hg38 reference genome, and reads were annotated with the GRCh38.84 annotation file. A gene expression matrix (count value) was generated with the “Digital Expression” command function. The raw data and processed gene expression matrix data were deposited in Genome Sequence Archive (GSA) for Humans under project PRJCA008156. The accession number is HRA001964.

**Analysis of polypeptide hormone-encoding genes**

Polypeptide hormone-encoding genes were collected from the Gene Expression Omnibus and published reports, and the genes with TPM＞2 in at least 30 cells were used for further clustering analysis.

**Immunohistochemical and immunofluorescence staining**

For immunofluorescence and immunohistochemistry staining, paraffin sections were cleared of paraffin, hydrated and “epitope-retrieved” in heated citrate buffer. The sections were blocked in 3% bovine serum albumin (BSA, Sigma-Aldrich) for 1 h at room temperature and then incubated with primary antibodies at 4 °C overnight, followed by incubation with secondary antibodies for 1 h at room temperature. For immunofluorescence staining, the nuclei were stained with 4′,6-diamidino-2-phenylindole (DAPI, Sigma-Aldrich). Immunohistochemistry staining was performed with an immunohistochemistry kit (ZSGB) following the manufacturer’s instructions, and the sections were counterstained with haematoxylin. Images were captured using a Zeiss LSM 780 confocal microscope. The immunofluorescence and immunohistochemistry staining were conducted using antibodies directed against HLA-G (Sigma-Aldrich), hCG (ZSGB), Adrenomedulin (Abcam), FSTL1 (Abcam), FSTL3 (Abcam), KISS1 (Abnova) and TAC3 (Abnova). Anti-rabbit Alexa Fluor 555 (Invitrogen) and anti-mouse Alexa Fluor 488 (Invitrogen) were used as secondary antibodies.

**Culture and differentiation of hTSCs**

Culture and differentiation of hTSCs were performed as our previously described[6]. Briefly, hTSCs were cultured in hTSCs medium, containing DMEM/F12 supplemented with 0.1 mM 2-mercaptoethanol, 0.2% FBS, 0.5% Penicillin-Streptomycin, 0.3% BSA, 1% ITS-X supplement, 1.5 mg/ml L-ascorbic, 50 ng/ml EGF, 2 μM CHIR99021, 0.5 μM A83-01, 1 μM SB431542, 0.8 mM VPA and 5 μM Y27632. To induce differentiation of hTSCs to STB, hTSCs were dissociated into single cells with TrypLE for 8 min at 37°C. Then, hTSCs were seeded in 6-well plates pre-coated with 2.5 mg/ml Collagen I at a density of 1 × 10^5^ cells per well and cultured in STB medium (DMEM/F12 supplemented with 0.1 mM 2-mercaptoethanol, 0.5% Penicillin-Streptomycin, 0.3% BSA, 1% ITS-X supplement, 2.5 μM Y27632, 2 μM forskolin, and 4% KSR). The medium was replaced every 2 days, and the cells were collected on day 6 for RT-PCR analysis. For the induction of EVT cells, hTSCs were seeded in a 6-well plate pre-coated with 1 μg/ml Collagen I at a density of 1 × 10^5^ cells per well and cultured in EVT medium 1 (DMEM/F12 supplemented with 0.1 mM 2- mercaptoethanol, 0.5% Penicillin-Streptomycin, 0.3% BSA, 1% ITS-X supplement, 100 ng/ml NRG1, 7.5 μM A83-01, 2.5 μM Y27632, 4% Knockout Serum Replacement (KSR) and 2% Matrigel) for 2 days. On day 2, the medium was replaced with the EVT medium 2, which eliminated NRG1 in EVT medium 1 and reduced the concentration of Matrigel to 0.25%, and cultured for 2 days. On day 4, replace the medium with EVT medium 3, which eliminated KSR from EVT medium 2, and cultured for 2 days. The cells were collected on day 6 for RT-PCR analysis.

To simulate the PE placenta undergoing a hypoxic environment, hTSC-derived STBs were incubated at 2% O_2_ for 72 hours.

**Real-Time Quantitative PCR (RT-PCR)**

Total RNA was isolated using a RNeasy Mini Kit (Qiagen). cDNA was synthesized using iScript Reverse Transcription Supermix kit (Bio-Rad) and amplified with SYBR Green PCR Master Mix (YEASEN) on a Touch Thermal Cycler Real-Time PCR system (Roche, LightCycler480). GAPDH was used as endogenous reference control. The primers for RNA quantification used in this study as follows:

RT-GAPDH-F, GGAGCGAGATCCCTCCAAAAT

RT-GAPDH-R, GGCTGTTGTCATACTTCTCATGG

RT-ADM-F, ATGAAGCTGGTTTCCGTCG

RT-ADM -R, GACATCCGCAGTTCCCTCTT

RT-FSTL1-F, GAGCAATGCAAACCTCACAAG

RT- FSTL1-R, CAGTGTCCATCGTAATCAACCTG

RT-ANGPTL4-F, GTCCACCGACCTCCCGTTA

RT-ANGPTL4-R, CCTCATGGTCTAGGTGCTTGT

RT-CSH1-F, ACAGAAACAGGTGGGGTCAAG

RT-CSH1-R, TTATTAGGACAAGGCTGGTGGG

RT-TAC3-F, ATGCTGCTATTCACAGCCATC

RT-TAC3-R, GGCTCCTTACAGACAGCCC

RT-FSTL3-F, GTGCCTCCGGCAACATTGA

RT-FSTL3-R, GCACGAATCTTTGCAGGGA

RT-PGF-F, GAACGGCTCGTCAGAGGTG

RT-PGF-R, ACAGTGCAGATTCTCATCGCC

RT-PAPPA-F, ACAAAGACCCACGCTACTTTTT

RT-PAPPA-R, CATGAACTGCCCATCATAGGTG

RT-PAPPA2-F, AGAATAAGCCTGGCGATTTTGG

RT-PAPPA2-R, GGCCTTAGGTAGTTCCCAGC

**Statistical analysis**

Two-tailed unpaired student’s t test was applied for analyzing experiment data after testing normal distribution. All histograms were plotted by GraphPad Prism 8 software, and *p*<0.05 was regarded as being significant for all the tests. All data were depicted as mean ± S.D.

**Data availability**

All sequencing data analyzed in this study have been deposited in Genome Sequence Archive (GSA) for Humans under project PRJCA008156. The accession number is HRA001964.

The following is the published datasets used in this study:

| **Author(s)** | **Year** | **Dataset title** | **Dataset URL** | **Database and identifier** |
| --- | --- | --- | --- | --- |
| Fan X, Liu Y,  Wang R | 2018 | Single-cell RNA-seq reveals the diversity of trophoblast subtypes and patterns of differentiation in the human placenta | https://www.ncbi.nlm.nih.gov/geo/query/acc.cgi?acc=GSE89497 | NCBI Gene Expression Omnibus, GSE89497 |
| Johan Henriksson, Mirjana Efremova Roser Vento Tormo | 2019 | Reconstructing the human first trimester fetal-maternal interface using single cell transcriptomics | https://www.ebi.ac.uk/biostudies/arrayexpress/studies/E-MTAB-6701?query=E-MTAB-6701 | ArrayExpress,  E-MTAB-6701 |
